# Major histocompatibility complex variation is similar in little brown bats before and after white‐nose syndrome outbreak

**DOI:** 10.1002/ece3.6662

**Published:** 2020-08-31

**Authors:** Xueling Yi, Deahn M. Donner, Paula E. Marquardt, Jonathan M. Palmer, Michelle A. Jusino, Jacqueline Frair, Daniel L. Lindner, Emily K. Latch

**Affiliations:** ^1^ Department of Biological Sciences University of Wisconsin‐Milwaukee Milwaukee WI USA; ^2^ Northern Research Station USDA Forest Service Rhinelander WI USA; ^3^ Northern Research Station USDA Forest Service Madison WI USA; ^4^ Department of Plant Pathology University of Florida Gainesville FL USA; ^5^ Roosevelt Wild Life Station SUNY College of Environmental Science and Forestry Syracuse NY USA

**Keywords:** fungal disease, immunity, major histocompatibility complex, *Myotis lucifugus*, North American bat, *Pseudogymnoascus destructans*

## Abstract

White‐nose syndrome (WNS), caused by the fungal pathogen *Pseudogymnoascus destructans* (Pd), has driven alarming declines in North American hibernating bats, such as little brown bat (*Myotis lucifugus*). During hibernation, infected little brown bats are able to initiate anti‐Pd immune responses, indicating pathogen‐mediated selection on the major histocompatibility complex (MHC) genes. However, such immune responses may not be protective as they interrupt torpor, elevate energy costs, and potentially lead to higher mortality rates. To assess whether WNS drives selection on MHC genes, we compared the MHC *DRB* gene in little brown bats pre‐ (Wisconsin) and post‐ (Michigan, New York, Vermont, and Pennsylvania) WNS (detection spanning 2014–2015). We genotyped 131 individuals and found 45 nucleotide alleles (27 amino acid alleles) indicating a maximum of 3 loci (1–5 alleles per individual). We observed high allelic admixture and a lack of genetic differentiation both among sampling sites and between pre‐ and post‐WNS populations, indicating no signal of selection on MHC genes. However, post‐WNS populations exhibited decreased allelic richness, reflecting effects from bottleneck and drift following rapid population declines. We propose that mechanisms other than adaptive immunity are more likely driving current persistence of little brown bats in affected regions.

## Graphical abstract

We tested the hypothesis of white‐nose syndrome (WNS) selection on MHC genes of hibernating bats by comparing the MHC class II *DRB* gene in little brown bats sampled from sites having different exposure histories to Pd, the fungus that caused WNS. We compared the WNS‐naive population in Wisconsin and the multi‐year post‐WNS population in New York and Vermont and found similar MHC variation, including the same most common alleles, comparable allelic diversity, and insufficient genetic differentiation, all indicating the lack of WNS selection on MHC genes. Our results indicated that the studied little brown bat populations may not rely on adaptive immunity to persist under WNS, and other mechanisms such as the physiological and behavioral changes found in recent studies may be more effective under WNS.

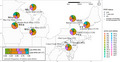

## 1 INTRODUCTION

Emerging and invasive fungal diseases represent an increasing threat to biodiversity worldwide (Fisher et al., 2012). Many fungal pathogens are host generalists and some can be highly virulent, resulting in massive population declines across multiple species. In addition, fungal pathogens are opportunistic with infections facilitated by environmental fluctuations and cross‐continental anthropogenic movements (Fisher et al., 2012). These characteristics make it challenging to prevent large‐scale infection by fungal pathogens across communities. Efforts to mitigate these large‐scale infections may benefit from better understanding of host responses and potential adaptation to fungal pathogens.

Immune responses are important defensive mechanisms used by animal hosts against fungal pathogens. Immunity of vertebrates involves two major parts: the first‐line innate immunity (e.g., skin barrier, inflammation, cytokine release) and the downstream adaptive immunity (function of B and T cells; Romani, 2004). Innate immunity is important to build the physical barrier, quickly recognize the pathogen, and initiate adaptive responses. Adaptive immunity is necessary to provide pathogen‐specific defenses including humoral responses using antibodies and cellular responses using helper T cells (e.g., Th1 and Th17 pathways; Blanco & Garcia, 2008; Verma, Wüthrich, Deepe, & Klein, 2015). Both antigen recognition and the initiation of adaptive responses require a group of immune receptors, the major histocompatibility complex (MHC) molecules, which bind to and present specific pathogen fragments to T cells (Bernatchez & Landry, 2003; Spurgin & Richardson, 2010). Specifically, MHC class II molecules are responsible for extracellular pathogens, including fungi. Pathogen‐mediated selection on MHC genes has been widely studied across taxa (Bateson, Whittingham, Johnson, & Dunn, 2015; Eizaguirre, Lenz, Kalbe, & Milinski, 2012; Kyle et al., 2014; Loiseau et al., 2009; Mainguy, Worley, Coˆté, & Coltman, 2007; Rico et al., 2016; Rico, Morris‐Pocock, Zigouris, Nocera, & Kyle, 2015; Strand et al., 2012; Sutton, Robertson, & Jamieson, 2015; Wegner, Reusch, & Kalbe, 2003; Zhang, Wu, Hu, Wu, & Wei, 2015). Recent studies found selection on MHC genes by the fungal pathogen Bd (*Batrachochytrium dendrobatidis*), which causes chytridiomycosis in amphibians (Bataille et al., 2015; Kosch et al., 2018; Meurling, Siljestam, Ritcher‐Boix, Laurila, & Hoglund, 2019; Savage & Zamudio, 2011, 2016), supporting the function of MHC molecules and adaptive immunity in defense to fungal diseases. The establishment of adaptive immunity is critical for host species to develop long‐term resistance and achieve co‐existence equilibrium with pathogens.

White‐nose syndrome (WNS) is an infectious fungal disease affecting North American hibernating bats. WNS is caused by an invasive fungal pathogen *Pseudogymnoascus destructans* (Pd) which originated in Europe and presumably colonized North America through anthropogenic transport (Leopardi, Blake, & Puechmaille, 2015; Lorch et al., 2011; Minnis & Lindner, 2013; Warnecke et al., 2012). European bats appear to be more tolerant to Pd, through long‐term co‐existence with the fungus, than North American bats, which were exposed to Pd for the first time in 2006 and have since been experiencing alarming mortality and population declines (Zukal et al., 2016). Pd grows on the skin of hibernating bats and erodes the epidermis, which initiates a cascade of physiological changes that lead to more frequent arousal from torpor, followed by increased dehydration, premature depletion of fat reserves, and consequently mortality of infected individuals (Blehert et al., 2009; Verant et al., 2014; Warnecke et al., 2012). Since its initial discovery in New York, WNS has caused large‐scale declines in many bat species (Frick et al., 2015), notably the little brown bat (*Myotis lucifugus*). Early on it appeared that the little brown bat, once one of the most common bats in North America, was in danger of widespread extirpation by WNS (Blehert et al., 2009; Frick et al., 2010). However, large numbers of little brown bats may have buffered populations from local extinction (Frick et al., 2015), providing an opportunity for adaptation under Pd‐mediated selection. In fact, little brown bats in New York have persisted after years of WNS infection in spite of continued Pd exposure (Cheng et al., 2019; Dobony et al., 2011; Langwig et al., 2017).

Multiple mechanisms have been proposed for population persistence in the face of Pd (Cheng et al., 2019), including environmental refugia (Flory, Kumar, Stohlgren, & Cryan, 2012; Langwig et al., 2012; Verant, Boyles, Waldrep, Wibbelt, & Blehert, 2012) and host tolerance or resistance to infection. Tolerance mechanisms reduce mortality without clearing the fungal infection, such as through altered hibernation behavior (Reeder et al., 2012) or increased fat storage (Cheng et al., 2019). In contrast, resistance mechanisms clear infection and prevent fungi growth with help from the immune system, such as the skin barrier and stronger healing capacity (Harazim et al., 2018), and the adaptive immunity with Pd antibodies (Davy et al., 2017; Donaldson et al., 2017; Field et al., 2015; Lilley et al., 2017; Moore et al., 2013; Rapin et al., 2014; Rocke et al., 2019). Tolerance and resistance mechanisms have different evolutionary implications and can result in different patterns of pathogen transmission (Baucom & Roode, 2011; Vander Wal et al., 2014). For example, a pathogen may quickly spread across populations of a tolerant host, but be prohibited from transmission among a resistant host due to their ability to suppress pathogen growth. Illuminating the specific mechanism(s) involved in bat population persistence is critical for understanding host–pathogen co‐existence and identifying effective conservation strategies.

Resistance via adaptive immunity has been hypothesized to occur in little brown bats based on their ability to mount a cascade of immune responses under Pd infection. Following epidermis erosion, innate immunity causes regional inflammation and cytokine release (Field et al., 2015; Lilley et al., 2017; Moore et al., 2013; Rapin et al., 2014), possibly initiating adaptive immunities such as cellular responses through Th17 pathway and humoral responses through Pd antibodies (Field et al., 2015; Lilley et al., 2017; Rocke et al., 2019). However, the degree to which such immune responses may be protective remains unclear. Bats are mostly infected by Pd during hibernation (Langwig et al., 2015) when their immune systems are normally restricted as a trade‐off of torpor (Bouma, Carey, & Kroese, 2010; Field et al., 2018). Mounted immune responses incur additional energy costs and more frequent arousals (Field et al., 2015; Lilley et al., 2017; Moore et al., 2013), two major drivers of mortality in hibernating bats (Reeder et al., 2012; Verant et al., 2014; Warnecke et al., 2012). Conversely, adaptive immunity could be protective by preventing fungal infection before bats go into deep torpor, a possibility underscored by successful vaccination of little brown bats using Pd antigens (Rocke et al., 2019). It remains unknown whether the protective immunity obtained from vaccination also evolved in natural populations persisting with Pd. If so, specific MHC genes encoding molecules that bind Pd fragments should be under strong directional selection, resulting in different MHC allele components between WNS naïve and persisting populations. Otherwise, similar MHC gene diversity in pre‐ and post‐WNS populations might indicate that MHC molecules are not selected under WNS and that mechanisms other than adaptive immunity may drive protection in natural populations of hibernating bats.

Potential WNS selection on the MHC class II *DRB* gene has been suggested for little brown bats (Davy et al., 2017; Donaldson et al., 2017), but interpretation of previous results has been limited by unidentified gene duplication and sparse post‐WNS sampling. The MHC gene is often characterized by copy number variation (CNV) both among species and among individuals within a species (Sommer, Courtiol, & Mazzoni, 2013). The majority of research examined 3 loci (2–6 alleles per individual) in bat species (Mayer & Brunner, 2007; Real‐Monroy, Martínez‐Méndez, & Ortega, 2014; Richman et al., 2010; Salmier, de Thoisy, Crouau‐Roy, Lacoste, & Lavergne, 2016; Schad, Dechmann, Voigt, & Sommer, 2011; Schad, Voigt, Greiner, Dechmann, & Sommer, 2012). However, studies of the sac‐winged bat (*Saccopteryx bilineata*) identified 3 loci using the traditional method of PCR and cloning (Mayer & Brunner, 2007) but up to 10 loci using next‐generation sequencing (NGS; Schad et al., 2012), indicating higher power but also potentially increased errors using NGS methods (Lighten, Van Oosterhout, Paterson, McMullan, & Bentzen, 2014; Salmier et al., 2016; Sommer et al., 2013). Copy number variation of the *DRB* gene in the little brown bat remains unclear and needs to be described to facilitate interpretation of gene diversity patterns. In addition, the previous study only compared individuals that succumbed after one‐year exposure to Pd against WNS‐naïve individuals sampled 20 years ago (Davy et al., 2017), making it unclear whether the detected difference in MHC allelic diversity was due to drift or adaptation. Therefore, additional study is needed to address the effect of WNS on MHC gene diversity in contemporary little brown bats having different Pd exposure histories.

Here, we tested whether WNS has produced selection on the MHC class II *DRB* gene in little brown bats after multi‐year exposure to Pd. We focused on exon 2 sequences which encode peptide binding regions and have commonly been studied (Davy et al., 2017). We first identified copy number variation of the *DRB* gene using NGS data, and then, we compared MHC polymorphism across populations having different Pd exposure histories. We aimed to test if pre‐ and post‐WNS populations had different MHC allelic diversity, such as the previously detected increase of rare alleles in post‐WNS populations (Davy et al., 2017). Changes of MHC allelic diversity following WNS outbreaks, especially in multi‐year persisting populations, would be consistent with WNS selection on MHC genes and could indicate evolution of effective adaptive immunity against Pd infection in wild populations.

## 2 MATERIALS AND METHODS

### 2.1 Sampling and DNA extraction

**FIGURE 1:**
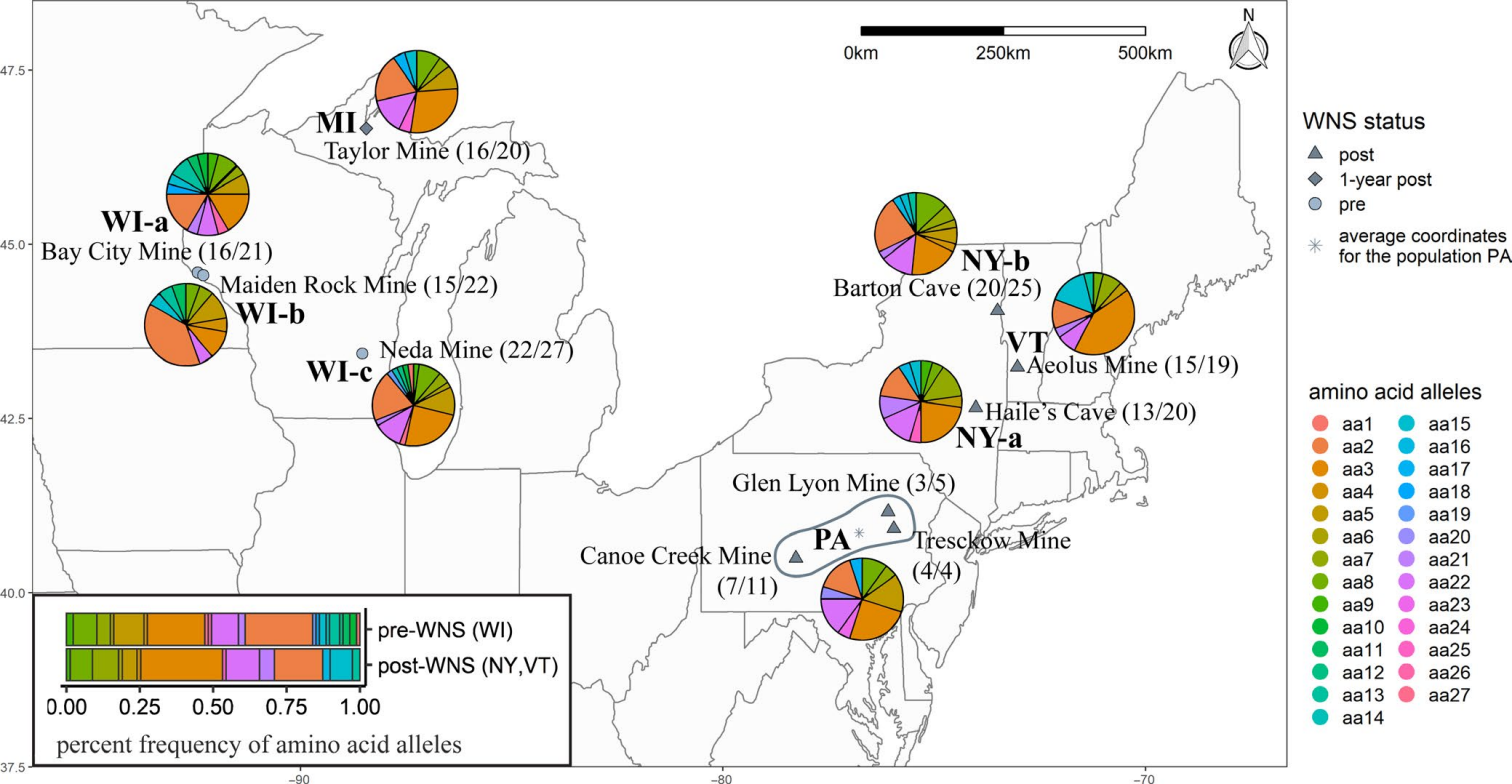
Distribution of sampling sites and the amino acid alleles. The 10 sampling locations are marked with symbols that indicate WNS infection status and labeled with site names and sample sizes (in parentheses, # successfully genotyped samples/ # collected samples). Pie charts indicating percent amino acid allele frequencies were placed around their corresponding sites. Site PA was a combination of three sampling locations with their average coordinates (mapped) used as the population coordinates. A bar chart (bottom left) shows the percent frequency of amino acid alleles between pre‐ (WI‐a, WI‐b, WI‐c) and post‐WNS (NY‐a, NY‐b, VT) populations

Wing tissue samples of little brown bats were collected during 2014 to 2015 from ten different hibernacula sites across five states (Figure 1; U.S. Forest Service Institutional Animal Care & Use, IACUC 2016‐001). At the time of our sampling, Wisconsin bats were not yet exposed to Pd, Michigan bats had been exposed for one year, and New York, Vermont, and Pennsylvania bats had been exposed to Pd for multiple years (around 5–7). Samples were mostly collected in April and May before spring emergence, except in MI (collected in November due to limited accessibility). We targeted a minimum of 20 individuals per sampling site, but the three sites in Pennsylvania each had very few samples available due to WNS‐induced mortality. To guarantee relatively even sample sizes for calculating genetic statistics, all PA hibernacula were combined as one sampling site and their coordinates averaged as the spatial location of the site (Figure 1). Individuals from all of our sampling sites were assumed to be from the same genetic population based on the previously characterized widespread gene flow and limited genetic structure of little brown bat populations (Vonhof, Russell, & Miller‐Butterworth, 2015), although gene flow may be reduced in the post‐WNS population due to the decreased population size.

Bats were captured in cooperation with the corresponding state natural resource agencies and in conjunction with planned hibernaculum monitoring, following the outlined safe handling protocols in the American Society of Mammologists (Sikes & Gannon, 2011). Tissues were extracted from plagiopatagium membranes with 2 or 4 mm sterile biopsy punches (Worthington, 1996) and were stored in 2‐ml tubes at −20°C. DNA was extracted using the Qiagen DNeasy^®^ Blood and Tissue Kit following the manufacturer's protocols.

### 2.2 Next‐generation sequencing and preprocessing

BLAST searches and Ensembl tools were used to identify little brown bat MHC II *DRB* paralogs from the reference genome (Myoluc2.0, GenBank GCA_000147115.1) with Ensembl annotation database (release version 73; Zerbino et al., 2017). To target exon 2 and to avoid amplification bias, two forward primers were modified from the EX2F primer which was originally developed for other *Myotis* species (Richman et al., 2010); one universal reverse primer was designed based on a conserved block of sequence extending slightly into the end of exon 2 (Table S1). Barcode sequences for individual identification were linked to the universal reverse primer. Primers were fused with Ion P1 and Ion A adapters for sequencing on the Ion Torrent PGM platform, according to manufacturer's instructions. To avoid PCR error from using multiple primers at the same site, each sample was amplified in two separate reactions using one of the forward primers and the universal reverse primer (Kanagawa, 2003). Each reaction was performed in a total volume of 20 μl containing 5U Platinum *Taq* Polymerase (Invitrogen), 2 μl 10× PCR Buffer, 0.4 μM each primer, 0.2 mM dNTPs, 2 mM MgCl_2_, and 2–20 ng template DNA. Thermal cycling was performed as 4 min at 94°C followed by 28 cycles of 30 s at 94°C, 45 s at 58°C, and 90 s at 72°C. PCR products were visualized on a 1.4% agarose gel stained with ethidium bromide, and gel band intensities were used to equally combine the two PCR products of the same sample. The combined PCR products were purified using 0.8% CloneWell gels and the E‐gel system, and were subsequently quantified using the Qubit High Sensitivity DNA assay (Thermo Fisher). Purified PCR products of all samples were pooled in equimolar concentrations and were sequenced on a 314v2 chip using Ion PGM 400 bp Sequencing Kit, according to manufacturer's recommendations (Thermo Fisher).

Raw sequencing data from the Ion Torrent Server were obtained using the BaseCaller option [‐‐disable‐all‐filters], which kept intact barcodes and disabled 3ʹ quality filtering to prevent trimming of the reverse primer. The obtained unaligned BAM file was transformed into the FASTQ format raw data using BedTools (Quinlan & Hall, 2010). Raw data were preprocessed in two different ways through either the AMPtk pipeline (Palmer, Jusino, Banik, & Lindner, 2018) or the jMHC program (Stuglik, Radwan, & Babik, 2011). Data preprocessed by both programs were genotyped using the following methods and generated very similar results, except that the AMPtk‐processed data kept nucleotide alleles that had shifted reading frames, which is unlikely to be true for alleles of the coding region (Sommer et al., 2013). Therefore, the jMHC‐processed data were considered more accurate and were presented in the main text. The AMPtk‐processed results and data are available in the Appendices S1 and S2 Tables S3‐S4.

Because MHC alleles have highly variable sequences and copy numbers, similar reads within one amplicon could be different true alleles (Stuglik et al., 2011). Therefore, we preprocessed the raw sequencing data by treating all different reads as separate variant sequences using the program jMHC (Stuglik et al., 2011). Raw sequencing data were filtered by quality (*Q* > 20) and length (200–300 bp) and were transformed into FASTA format using cutadapt and awk. The filtered data were processed in jMHC to select reads that contained valid barcodes and the entire primer sequences. Because jMHC assumes that single barcode is linked to the forward primer whereas in our data barcodes were linked to the reverse primer, jMHC demultiplex was processed using the “2‐sided TAGs” command with the 5ʹ ends of forward primers input as “forward barcodes”. The raw sequencing data and TAG files input for jMHC program are available from the Dryad Digital Repository (https://doi.org/10.5061/dryad.76hdr7ssq). jMHC output the demultiplexed reads (barcodes and primers trimmed) together with their counts; these reads were further filtered in each sample independently using R 3.5.2 (R Core Team, 2018) and Geneious 10.0.6 (https://www.geneious.com/). Samples with the total depths (read counts) lower than 500 were excluded due to failure of amplification. For each remaining sample, reads were removed from analyses if they had only one count (singletons) or shifted reading frames (Sommer et al., 2013). Lastly, a summary table was generated to show the counts of remaining reads (i.e., variant sequences) in each successfully processed sample.

### 2.3 Identification of putative alleles

Variant sequences in the preprocessed data contained putative alleles but also artifacts and PCR errors. To identify putative alleles in each sample (i.e., to genotype), we applied the CNV‐DOC method (Bateson et al., 2015; Lighten et al., 2014), which calls alleles using two different models, namely the copy number variation (CNV) and the degree of change (DOC). Under the assumption that true alleles always have higher depths of reads than artifacts or errors, the CNV‐DOC method picks out the 10 most abundant variant sequences within each sample and compares their observed depths with the expected depths under different scenarios of allele numbers. For each sample, the CNV model outputs the two best‐fit scenarios of allele numbers, and the DOC model outputs one most likely allele number together with a DOC score that indicates model reliability (Lighten et al., 2014). In most of our samples, at least one CNV output equaled the DOC output, and we used this as the estimated allele number. When the CNV and DOC outputs differed, we used the CNV estimate if the two CNV estimates were the same and the DOC output was less reliable (DOC score < 50). Otherwise, the number of alleles was undetermined and the sample was removed from the analyses. Based on the estimated number of alleles in each sample, the most abundant variant sequences were extracted from their demultiplexed reads and translated in Geneious 10.0.6 using frame 1. Sequences translated into premature stop codons were removed from analysis, together with the samples in which these variants were found. The remaining variant sequences were regarded as the identified nucleotide alleles. A Spearman correlation was constructed between the total depth and the number of identified nucleotide alleles in each sample to test if the allele number was related to the sequencing depth.

Translations of the nucleotide alleles were processed on CD‐HIT Suite (http://weizhong‐lab.ucsd.edu/cdhit‐web‐server/cgi‐bin/index.cgi?cmd=cd‐hit; Huang, Niu, Gao, Fu, & Li, 2010) to remove duplicates and to generate the set of amino acid alleles. The amino acid alleles identified in this study were aligned using Geneious 10.0.6 with previously published sequences of *DRB* exon 2 from other bat species, including *Myotis velifer* (Richman et al., 2010), *M. vivesi* (Richman et al., 2010), *Noctilio albiventris* (Schad et al., 2012), *Saccopteryx bilineata* (Schad et al., 2012), *Carollia perspicillata* (Schad et al., 2012), *Desmodus rotundus* (Salmier et al., 2016), and *Artibeus jamaicensis* (Real‐Monroy et al., 2014). Codons of the antigen‐binding sites were identified based on previous publications (Richman et al., 2010; Salmier et al., 2016). Antigen‐binding sites are considered to be under selection during evolution, leaving mainly nonsynonymous substitutions in these regions. Tests for synonymous versus nonsynonymous substitutions were conducted in MEGA7 (Kumar, Stecher, & Tamura, 2016) using the codon‐based Z‐test (Jukes‐Cantor, Nei‐Gojobori method; Nei & Gojobori, 1986) and the maximum likelihood analysis of natural selection (HyPhy package, Felsenstein 1981 model). *Z*‐test estimates difference between the number of synonymous substitutions per site (d*S*) and the number of nonsynonymous substitutions per site (d*N*) under the null hypothesis of strict neutrality (d*N*–d*S* = 0; Kumar et al., 2016). The HyPhy package in MEGA7 calculates d*S* and d*N* in a codon‐by‐codon manner, and the relative ratio of d*N*/d*S* was estimated by dividing the sum of dN by the sum of dS across antigen‐binding sites, nonantigen‐binding sites, and entire sequences.

### 2.4 MHC diversity and differentiation

Diversity of nucleotide alleles per sampling site was estimated in Arlequin 3.5.2 (Excoffier & Lischer, 2010) using the number of polymorphic sites, allelic richness (Theta *k*), mean number of pairwise differences (*π*), nucleotide diversity, and gene diversity. Diversity of amino acid alleles was evaluated based on the total number of alleles at each sampling site and the average number of alleles per individual. Amino acid allelic diversity was visualized using percent frequencies calculated as the number of individuals carrying an allele divided by the sum of allele numbers of all individuals from that sampling site. To test whether allelic diversity is related to sample size, we estimated Pearson correlation between the log‐transformed sample size and the number of alleles or allelic richness at each sampling site. To better evaluate the effect of WNS, samples were further pooled into pre‐ versus post‐WNS populations and their nucleotide allelic richness and amino acid allelic diversity were estimated using the above methods. Although MI and PA sites were considered post‐WNS, MI site was only one‐year post exposure, which was too short for detecting signals of adaption, and PA site might be biased by extremely small sample sizes from each hibernacula. Therefore, to detect the biggest possible effect, we only present the comparison between extreme cases of pre‐WNS population in WI and multi‐year post‐WNS population in NY and VT. Comparisons including samples from MI and PA are available in the Appendices S1.

Due to the unknown number of MHC loci in the little brown bat, the level of heterozygosity could not be calculated and the genetic structure could not be estimated using fixation indices such as *F*_ST_ or *G*_ST_ (Bateson et al., 2015). Instead, differentiation among sampling sites was measured by Jost's *D* (Jost et al., 2018) calculated in SpadeR (Chao, Ma, Hsieh, & Chiu, 2016) with an input of the number of samples carrying each nucleotide allele at each sampling site. To test whether genetic differentiation was shaped by limited geographic gene flow, patterns of isolation by distance (IBD) were analyzed using Mantel tests in the R package vegan (Jari Oksanen et al., 2018; Jombart, 2008). Mantel tests estimate the correlation between two matrices, genetic distance (Jost's *D*), and geographic distance (Euclidean), to control for associations due simply to the spatial effects. A partial Mantel test was applied to further control for sample size using the Bray–Curtis dissimilarity matrix calculated in the package vegan (Jari Oksanen et al., 2018). The Discriminant Analysis of Principal Components (DAPC) was carried out to visualize potential genetic differentiation based on the contribution of MHC alleles in the R package adegenet (Jombart, 2008; Jombart & Ahmed, 2011; Jombart, Devillard, & Balloux, 2010). DAPC maximizes between‐population variation while minimizing within‐population variation to sort out differentiation. Because ploidy levels of the MHC gene possibly vary among individuals, DAPC could not be carried out on the individual level but was computed to show the differentiation of identified alleles among sampling sites and populations. Individual nucleotide base pair was treated as different loci, and only polymorphic loci (50 nucleotide loci) were input for DAPC analysis. In DAPC of the 8 sampling sites, 13 principal components (PCs) and 5 discriminant functions were retained, corresponding to 90.4% of the conserved variance. In DAPC between pre‐ (WI) and post‐WNS (NY, VT) populations, 14 PCs and 1 discriminant function were retained, corresponding to 90.7% of the conserved variance. DAPC‐estimated membership probabilities were used to show the level of admixture among sampling sites.

## 3 RESULTS

### 3.1 Sampling and identification of MHC alleles

In total, 174 individuals were sampled and sequenced. The jMHC program successfully preprocessed 136 samples and all of them were successfully genotyped by the CNV‐DOV method; however, 4 putative alleles were translated into premature stop codons, making them unlikely to be true alleles, and they were all identified as the single allele in 5 samples. Therefore, these 4 alleles and the corresponding 5 samples were removed from analysis, leaving 131 successfully genotyped individuals with 45 nucleotide alleles and 27 amino acid alleles. We identified 1 to 5 alleles (average 2 alleles) per individual, suggesting up to 3 loci of the *DRB* gene in the little brown bat (Table 1) and in line with observations of 1 to 3 loci in previously studied bat species (Mayer & Brunner, 2007; Real‐Monroy et al., 2014; Richman et al., 2010; Salmier et al., 2016; Schad et al., 2011, 2012), except for the sac‐winged bat which was identified with 10 loci (Schad et al., 2012). A significant but weak correlation (*R* ^2^ = 0.11, *p* < .01) was detected between the sequencing depth and the number of nucleotide alleles (Figure S1), suggesting dropout of low‐depth putative alleles due to differential amplification efficiency. However, the small correlation coefficient indicated that potential allelic dropout was modest and that our results should cover most of the putative alleles in the studied populations.

**TABLE 1:** Summary of little brown bat MHC diversity across sampling sites and in the pooled pre‐ and post‐WNS populations

Sampling site	*n*	Nucleotide alleles								Amino acid alleles		
		*I*_N_ (*R*_N_)	*A*_N_	*U*_N_	# Polymorphic sites	Theta k (95% CI)	*π*	Nucleotide diversity	Gene diversity	*I*_A_ (*R*_A_)	*A*_A_	*U*_A_
Post‐WNS												
NY‐a	13	1.69 (1–3)	14	3	21	15.53 (6.96, 35.31)	4.38	0.016	0.94	1.69 (1–3)	11	1
NY‐b	20	1.55 (1–2)	16	4	23	12.59 (6.33, 24.99)	4.06	0.014	0.93	1.55 (1–2)	12	0
VT	15	1.73 (1–4)	11	1	17	6.66 (3.08, 14.11)	4.05	0.014	0.83	1.73 (1–4)	9	0
PA	14	1.50 (1–2)	11	3	13	8.61 (3.80, 19.44)	3.45	0.012	0.91	1.43 (1–2)	9	2
MI^a^	16	1.31 (1–2)	13	3	17	13.57 (6.00, 31.19)	3.94	0.014	0.91	1.31 (1–2)	9	1
NY and VT	48	1.64 (1–4)	22			9.76 (5.81, 16.05)				1.64 (1–4)	14	
Pre‐WNS												
WI‐a	16	1.50 (1–3)	16	3	21	19.81 (9.09, 44.28)	4.10	0.015	0.96	1.50 (1–3)	14	2
WI‐b	15	1.27 (1–3)	14	2	22	22.46 (9.20, 57.48)	3.51	0.012	0.97	1.20 (1–3)	10	0
WI‐c	22	2.14 (1–5)	21	8	40	14.01 (7.83, 24.80)	4.39	0.016	0.93	2.05 (1–5)	15	5
WI	53	1.70 (1–5)	29			14.43 (9.07, 22.64)				1.64 (1–5)	21	
Total	131	1.61 (1–5)	45							1.58 (1–5)	27	

Note

*n*: number of genotyped individuals; *I_N_* (*R_N_*): average (range) number of nucleotide alleles per individual; *A_N_*: number of different nucleotide alleles from the site or population; *U_N_*: number of unique nucleotide alleles from the site; Theta *k*: index of allelic richness (95% confidence interval); *π*: mean number of pairwise differences; *I*_A_ (*R*_A_): average (range) number of amino acid alleles per individual; *A*_A_: number of different amino acid alleles from the site or population; *U*_A_: number of unique amino acid alleles from the site.

^a^ The MI samples were collected after 1 year of exposure to Pd.

**FIGURE 2:**
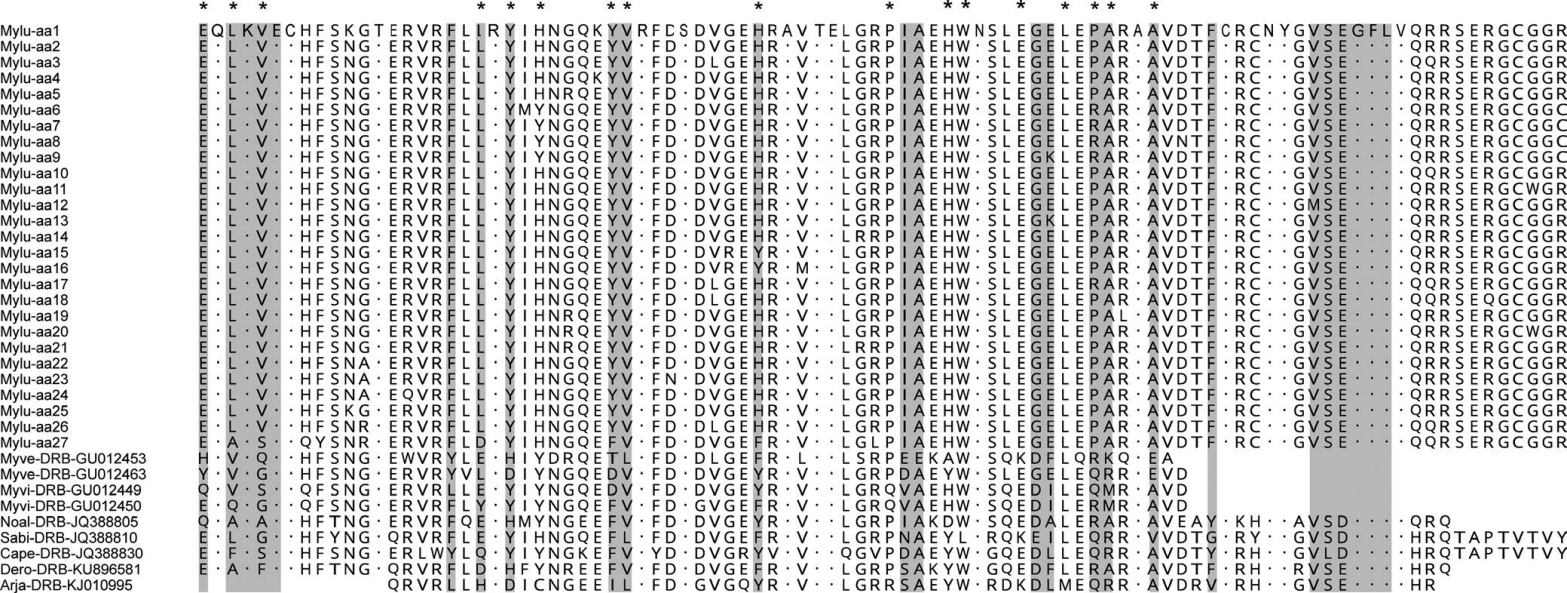
Alignment of amino acid alleles and identification of antigen‐binding sites in MHC *DRB* exon 2. The 27 amino acid alleles of little brown bat identified in this study were aligned with published sequences of *DRB* exon 2 in several other bat species (GenBank accession numbers given in the sequence names). Two sets of antigen‐binding sites were identified, the * codons (Richman et al., 2010) and the shaded codons (Salmier et al., 2016), based on different previous studies

By aligning the amino acid alleles with published MHC *DRB* sequences in other bat species (Figure 2), two sets of antigen‐binding sites were identified based on two previous studies, resulting in either 17 (*) (Richman et al., 2010) or 25 (shaded) (Salmier et al., 2016) codons of antigen‐binding sites. *Z*‐tests on both sets of antigen‐binding sites failed to reject the null hypotheses of neutrality despite elevated d*N* values: d*N*–d*S* = 0.269 (*p* = .789) for the 17 codons, and d*N*–d*S* = 0.080 (*p* = .936) for the 25 codons. Similarly, maximum likelihood analyses were nonsignificant under the null hypotheses of neutrality. The estimated d*N*/d*S* ratio was 0.378 across all 94 codons. The estimated d*N*/d*S* ratio of antigen‐binding sites was close to one but was much higher than the ratio of nonantigen‐binding sites: 0.82 for the 17 codons (0.29 for the remaining 77 codons of nonantigen‐binding sites) and 1.07 for the 25 codons (0.23 for the remaining 69 codons of nonantigen‐binding sites). Accordingly, although we failed to detect significance of the d*N*/d*S* ratio in antigen‐binding sites, the relative rate of nonsynonymous substitution was higher in antigen‐binding sites compared to that in nonantigen‐binding sites or across the whole sequences. The lack of significance in d*N*/d*S* ratio was probably due to the lack of power in our tests using samples from genetically admixed populations, in which case the d*N*/d*S* ratio mainly reflects short‐term polymorphism segregation rather than long‐term substitution during evolution (Kryazhimskiy & Plotkin, 2008; Richman et al., 2010). In addition, the d*N*/d*S* ratio may not be significantly elevated if the putative antigen binding site is buried inside the protein rather than located on the surface and exposed to the outside. Further analysis is required to test whether the antigen‐binding sites have different locations and whether their exposure levels result in different d*N*/d*S* ratios.

### 3.2 MHC diversity and population structure

**FIGURE 3:**
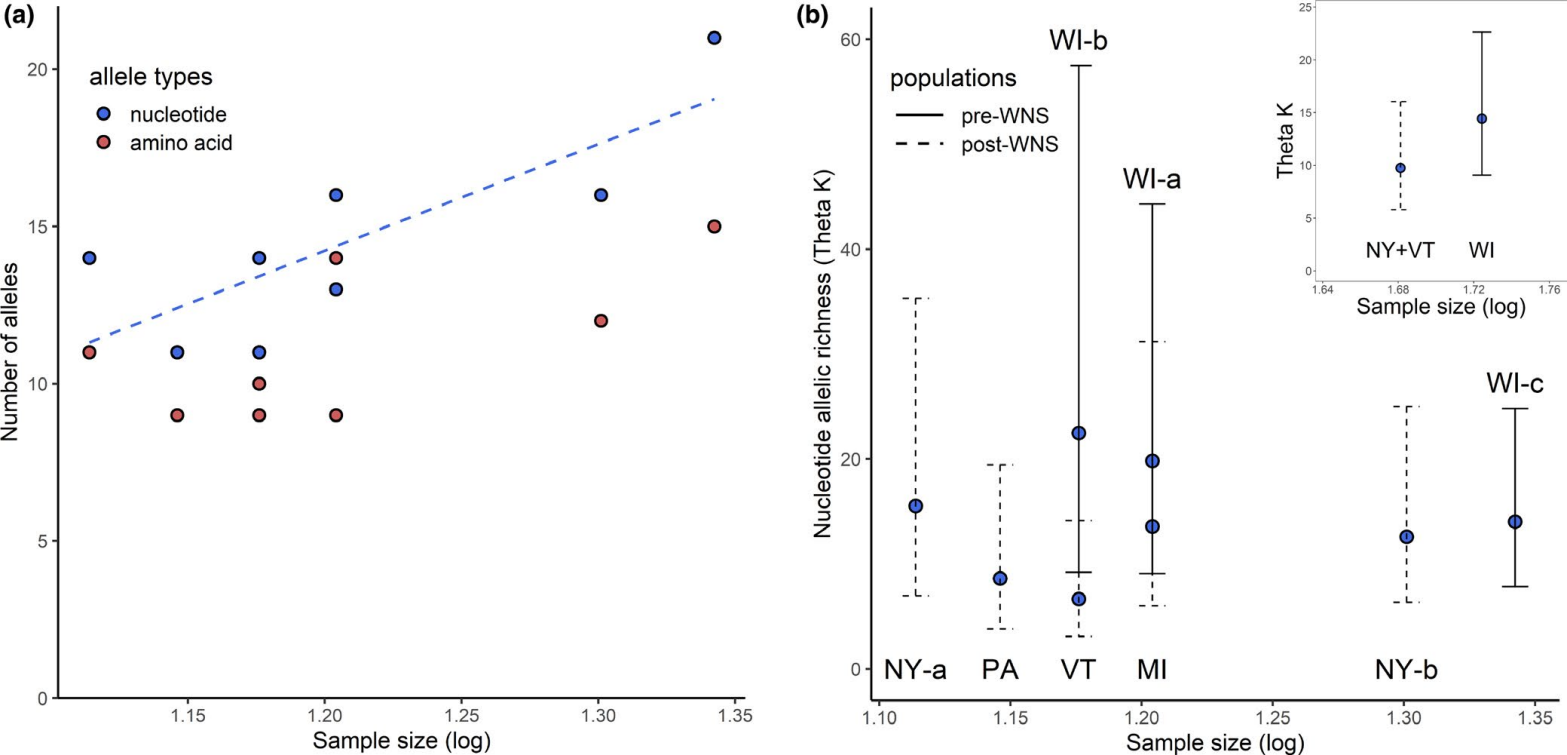
Relationship between sample size and (a) number of alleles or (b) allelic richness (Theta *K*). The best‐fit linear line shows significant relationship between sample size and the number of nucleotide alleles. Error bars of Theta *K* show 95% confidence intervals and line types of error bars indicate WNS infection status of the corresponding sites. Theta *K* against sample size for the grouped pre‐ and post‐WNS populations was also included in (b)

Diversity indices of nucleotide alleles were summarized in Table 1. A significant Pearson correlation (*r* = .802, *p* = .017) was detected between the number of identified nucleotide alleles and the log‐transformed sample size (Figure 3a). Therefore, to compare nucleotide allelic diversity across sampling sites, we focused on the index of allelic richness (Theta *K*) calculated by controlling for sample size (*r* = .001, *p* = .998, Figure 3b). Sites WI‐a and WI‐b had the highest Theta *K* while PA and VT had the lowest Theta *K* (Table 1), showing variation of nucleotide diversity across the study area. Consistently, higher allelic richness was detected in the pooled pre‐WNS population (WI) compared to the pooled post‐WNS population (NY, VT), but the difference was weak and nonsignificant with highly overlapped 95% confidence interval (Table 1, Figure 3b). Diversity of amino acid alleles was summarized in Table 1 and visualized in Figure 1. No significant effect of sample size was detected for the amino acid alleles (*r* = .667, *p* = .071, Figure 3a), suggesting a good coverage of amino acid diversity in our samples. The number of amino acid alleles was highest in sites WI‐a and WI‐c and lowest in sites PA, VT, and MI (Table 1). Relatively more amino acid alleles were identified in the pre‐WNS population (WI) compared to the post‐WNS population (NY, VT, Table 1). Results of the amino acid diversity and nucleotide allelic richness (Theta *K*) were very similar when MI and PA samples were involved in the post‐WNS population (Figure S2). Accordingly, diversities of both nucleotide alleles and amino acid alleles showed variation across sampling sites, with somewhat higher allelic richness in the pre‐WNS sites (WI) and the lowest allelic richness in VT and PA.

**FIGURE 4:**
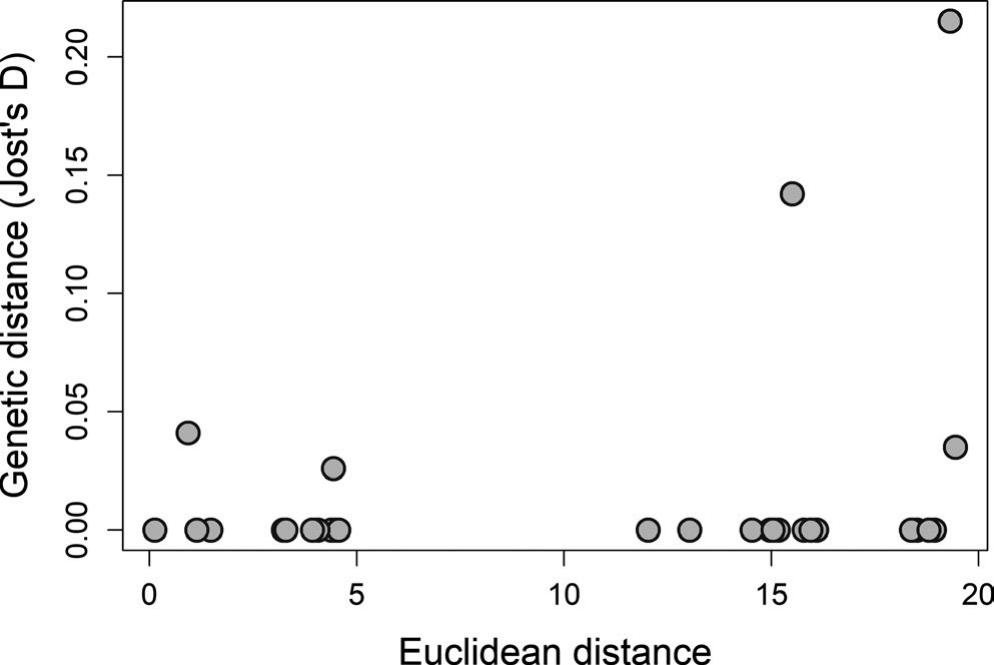
The test of isolation by distance. No significant relationships were detected by mantel tests (*p* > .05)

**FIGURE 5:**
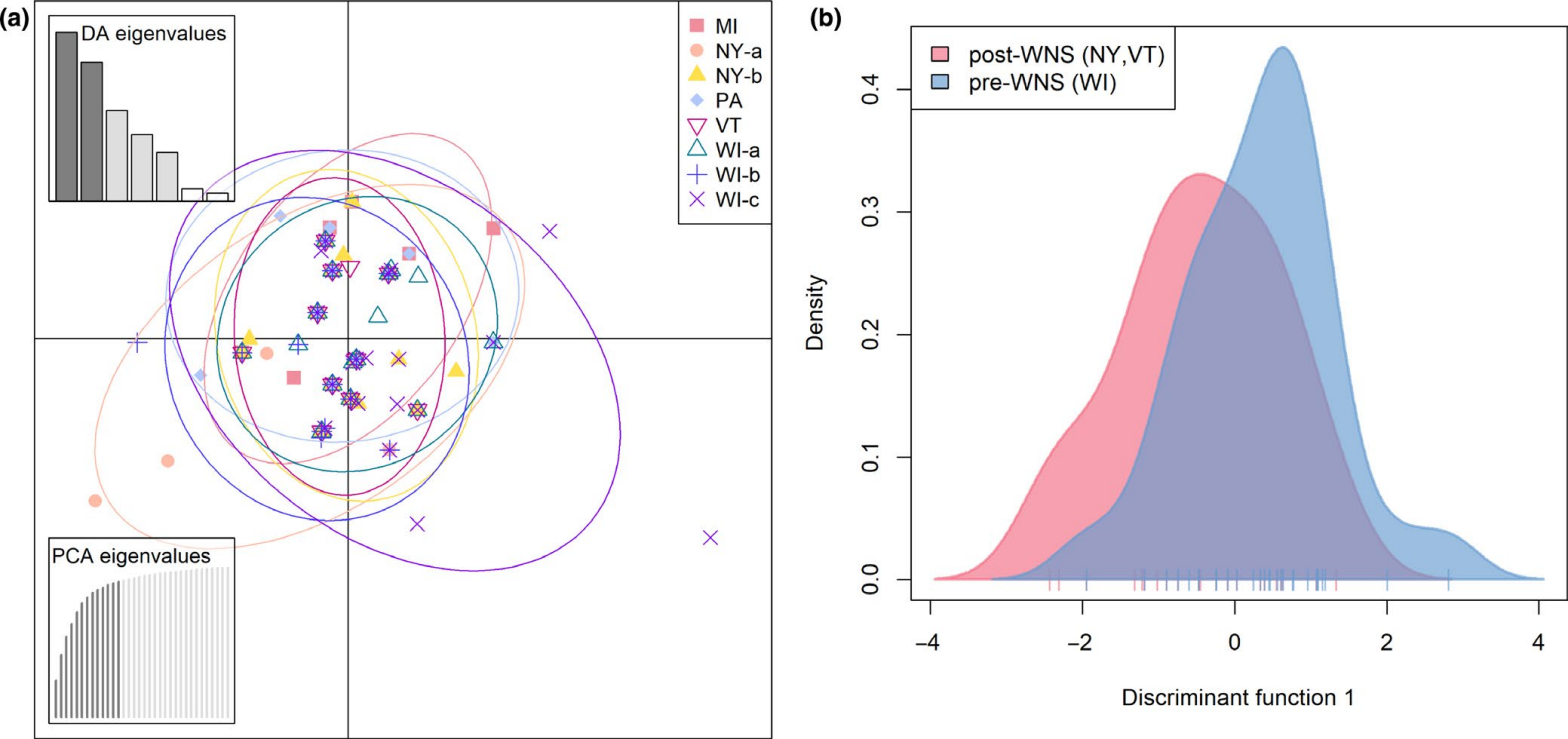
DAPC analysis across (a) all sampling sites and (b) the pre‐ and post‐WNS populations. The size of the inertia ellipse was set 2.5 in R package adegenet to encompass approximately 95% of the alleles. The sampling sites and populations were highly admixed and showed no spatial differentiation

Overall, no genetic structure at the *DRB* gene was detected across the studied region. Pairwise Jost's *D* showed no genetic differentiation (Table S2); VT was the only sampling site with positive mean pairwise Jost's *D* compared with all the other sites except MI and NY‐a, yet all the 95% confidence intervals of pairwise Jost's *D* overlapped and included zero, indicating a lack of genetic differentiation. No significant IBD was detected (alpha of 0.05) using either Mantel (*r* = .2536, *p* = .069, Figure 4) or partial Mantel tests (*r* = .243, *p* = .084). Results from DAPC showed high allelic admixture and a lack of genetic differentiation both among the sampling sites and between the pre‐ and post‐WNS populations (Figure 5). In addition, DAPC generated low posterior probabilities for reassigning alleles back to their sampling sites (Figure S3) with membership probabilities ranging from 0 (VT) to 0.524 (WI‐c) in the analysis of 8 sampling sites (overall mean 0.293), and 0.759 for the pre‐WNS and 0.545 for the post‐WNS populations in the analysis of two populations. These membership probabilities were insufficient for successful allele reassignments and thus indicated admixture. The above results consistently showed a lack of differentiation at the *DRB* gene across the studied area, regardless of the difference in Pd exposure histories.

## 4 DISCUSSION

The emerging fungal disease WNS has caused precipitous declines in North American hibernating bats, yet it is unclear whether WNS exerts selection on MHC genes in the infected populations. Here, we compared MHC class II *DRB* gene exon 2 across little brown bat populations varying in duration of Pd exposure (0–7 years) and did not find evidence of genetic differentiation. This result is similar to the previously demonstrated baseline MHC structure (Davy et al., 2017) and indicated a lack of WNS selection on MHC *DRB* gene in little brown bats.

The Pd‐naïve population and multi‐year Pd*‐*exposed population had comparable allelic diversities and the same most common amino acid alleles, a pattern that would not be expected if WNS exerted strong directional selection on certain MHC alleles, even if under some level of gene flow (Vonhof et al., 2015). In addition, the post‐WNS populations are expected to have a lower level of gene flow due to their greatly reduced population sizes. Accordingly, our data indicated limited roles played by MHC molecules in developing persistence under WNS in the studied populations. In other words, although Pd antibodies can be generated under infection (Field et al., 2015; Lilley et al., 2017; Rocke et al., 2019), the adaptive immune responses are probably not protective in hibernating bats and thus are not selected for by WNS in the infected little brown bat populations. This lack of WNS selection on MHC genes also supports a previous proposal that antibody‐mediated immune responses could not explain the survival of Pd‐infected bats in North America and Europe (Johnson et al., 2015). Similarly, a study found that a frog population maintained susceptible to chytridiomycosis despite the generation of *Bd*‐specific antibodies, suggesting ineffective adaptive immune responses to the fungal disease (Ellison et al., 2014). For hibernating bats, initiation of normally suppressed immune responses would interrupt torpor and deplete energy storage (Bouma et al., 2010; Field et al., 2015, 2018; Lilley et al., 2017; Moore et al., 2013; Rapin et al., 2014), both increasing the chance of mortality (Reeder et al., 2012; Verant et al., 2014; Warnecke et al., 2012). Upon the initial infection by Pd, the innate and primary adaptive immune responses might incur energetic and survival costs such that few individuals can survive to establish secondary adaptive immunity, at least not on a short evolutionary timescale such as in our study. In comparison, adaptive immunity gained through vaccination (Rocke et al., 2019) is more likely protective because bats would go through primary adaptive responses and generate memory cells of Pd antibodies before entering torpor; so during hibernation, Pd infection would directly initiate the downstream adaptive immunity, and it would be less energetically costly for the bats. Such effective adaptive immunity may take more generations to establish in naturally evolving populations, consistent with the undetectable WNS selection on the *DRB* gene and presumably also other MHC genes in current bat populations. A recent study using whole‐genome sequencing data from little brown bats detected signals of WNS selection on only one immune gene, MASP1, while all the other candidate genes were involved in hibernation behavior or fat storage (Gignoux‐Wolfsohn et al., 2018), further supporting the lack of WNS selection on MHC genes and the function of alternative survival mechanisms (Lilley et al., 2020).

Our data showed relatively lower allelic diversity in the post‐WNS populations, especially in VT and PA, and we propose that this pattern is mainly shaped by the demographic processes of bottlenecks and genetic drift rather than selection by WNS. A strong effect of neutral processes on MHC gene diversity has been found in several previous studies across different taxa (Bateson et al., 2015; Strand et al., 2012; Sutton et al., 2015). For highly variable loci like the MHC gene, bottlenecks might have little impact on heterozygosity, but genetic drift during the decline of population size can cause substantial losses of rare alleles and allelic richness (Eimes et al., 2011; Sutton et al., 2015). In fact, demographic bottlenecks were observed in PA sites during sampling given the high rate of WNS‐related mortality and rarity of surviving bats (but see Lilley et al., 2020). Individuals from VT were proposed to have similar MHC allelic diversity as the nearby NY individuals based on high levels of regional gene flow (Vonhof et al., 2015); however, our data showed relatively lower allelic richness and a weak genetic distinction of the VT samples, similar to the lowest post‐WNS allele frequencies among VT individuals detected in another study (Gignoux‐Wolfsohn et al., 2018). These results suggest potential site‐specific selection or demographic processes acting on the genetic diversity of VT population, yet additional research is required to test this hypothesis. A previous study (Davy et al., 2017) found increased rare alleles in post‐WNS populations composed of little brown bats that succumbed in the second winter after Pd exposure. We propose that this previous study might have actually sampled individuals that were extirpated in the bottleneck, in which case they would have detected the lost allelic diversity rather than the remaining alleles in the bottlenecked population. Future studies using both MHC and neutral genetic markers will help to demonstrate the proposed demographic bottlenecks and their effects on MHC allelic diversity in post‐WNS populations.

According to the results in this study, we suggest that resistance through adaptive immunity is not the current mechanism of persistence in little brown bat populations affected by WNS. Other resistance or tolerance mechanisms related to physiological and behavioral changes may be more effective to increase present survival (Auteri & Knowles, 2020; Johnson et al., 2015; Lilley et al., 2020), such as through skin microbiome (Ange‐Stark et al., 2019; Hoyt et al., 2019), increased fat storage (Cheng et al., 2019), and altered hibernating behavior (Gignoux‐Wolfsohn et al., 2018; Reeder et al., 2012). These physiological and behavioral mechanisms may also help to give time for effective adaptive immunity to evolve in natural populations. Although we detected no signal of WNS selection on the MHC gene, the WNS‐induced mortality followed by demographic bottlenecks and drift probably resulted in a decrease of MHC allelic richness in the post‐WNS populations, which could affect population viability by reducing their adaptive potential to other pathogens. Further research is needed to test this hypothesis, but such a change might be tolerable in little brown bats, considering the high frequencies of most common MHC alleles and the signal of recoveries of allelic diversity in the persisting NY populations.

## 5 CONCLUSIONS

In this study, we found no signal of WNS selection on the MHC class II *DRB* gene in little brown bats. We propose that the mounted immune responses exhibited by little brown bats (and likely similar species) are not protective and that persisting populations probably rely more on other resistance or tolerance mechanisms such as stronger skin barrier, increased fat storage, and changed hibernation behavior. On the other hand, we found relatively lower MHC allelic richness in bottlenecked populations immediately following steep WNS‐induced mortality. This loss of allelic richness might further impact population viability by reducing the potential of adaptation to other pathogens and future environmental changes, raising the importance of maintaining population size under WNS, although the vagility of bats and high degree of intermixing observed in past studies indicate a potential for recovery of allelic diversity. Results of this study indicate that effective adaptive immunity against Pd might take a long time to evolve naturally in wild bat populations.

## CONFLICT OF INTEREST

No conflict of interest is declared by any of the authors of this manuscript.

## AUTHOR CONTRIBUTION

**Xueling Yi:** Data curation (equal); Formal analysis (lead); Methodology (equal); Software (lead); Visualization (lead); Writing‐original draft (lead); Writing‐review & editing (equal). **Deahn M. Donner:** Conceptualization (equal); Funding acquisition (lead); Project administration (equal); Resources (equal); Supervision (equal); Writing‐review & editing (equal). **Paula E. Marquardt:** Conceptualization (equal); Writing‐review & editing (equal). **Jonathan M. Palmer:** Data curation (equal); Formal analysis (equal); Methodology (equal); Software (equal). **Michelle A. Jusino:** Data curation (equal); Methodology (equal). **Jacqueline Frair:** Data curation (equal); Resources (equal); Writing‐review & editing (equal). **Daniel L. Lindner:** Methodology (equal); Resources (equal). **Emily Latch:** Conceptualization (equal); Supervision (lead); Validation (equal); Writing‐review & editing (equal).

## Supporting information

Appendix S1

Tables S3‐S4

Appendix S2

## Data Availability

Sample information and the AMPtk‐processed data are available in Appendices S1 and S2. The raw sequencing data, jMHC tag files, CNV‐DOC genotyping results, sequences of the identified alleles (nuclear and amino acid), and the R codes used in this study are available from the Dryad Digital Repository (https://doi.org/10.5061/dryad.76hdr7ssq). The raw sequencing data include 181 samples, 7 of which were sampled from Illinois and were not analyzed in this study.
