# Supplementary material for: Major histocompatibility complex variation is similar in little brown bats before and after white‐nose syndrome outbreak
Source: Ecol Evol. 2020 Aug 31;10(18):10031–43. doi: 10.1002/ece3.6662 (PMC7520216; doi:10.1002/ece3.6662)
Supplement: Supplementary file 1 — Appendix S1 [file ECE3-10-10031-s001.pdf]

Supplementary information for Yi et al. 2020

MHC variation is similar in little brown bats before and after white-nose syndrome outbreak

S1 Table. Sequences of the forward and reverse primers designed in this study for amplifying MHC class II *DRB* exon 2 in the little brown bat (*Myotis lucifugus*).

Forward primer 1	Mylu IntF_GC	5’- TGTCCCCGCRGCGCATTTCCTG-3’
Forward primer 2	Mylu IntF_AA	5’- TGTCCCCGCAGCAAATTTCCTG-3’
Reverse primer	Mylu Int2R	5’- GGGTGCTCCTCACAGCCCTGTG-3’

S2 Table. Pairwise Jost’s D of the sampling sites estimated using nucleotide alleles of MHC *DRB* exon 2 (mean in the top and 95% confidence interval in the bottom diagonal).

	MI	NY-a	NY-b	PA	VT	WI-a	WI-b	WI-c
MI	-	0	0	0	0	0	0	0
NY-a	(0, 0.417)	-	0	0	0	0	0	0
NY-b	(0, 0.351)	(0, 0.339)	-	0	0.041	0	0	0
PA	(0, 0.398)	(0, 0.368)	(0, 0.347)	-	0.026	0	0	0
VT	(0, 0.300)	(0, 0.366)	(0, 0.392)	(0, 0.397)	-	0.035	0.215	0.142
WI-a	(0, 0.431)	(0, 0.388)	(0, 0.369)	(0, 0.374)	(0, 0.438)	-	0	0
WI-b	(0, 0.510)	(0, 0.487)	(0, 0.453)	(0, 0.477)	(0, 0.616)	(0, 0.444)	-	0
WI-c	(0, 0.313)	(0, 0.307)	(0, 0.269)	(0, 0.267)	(0, 0.424)	(0, 0.326)	(0, 0.413)	-

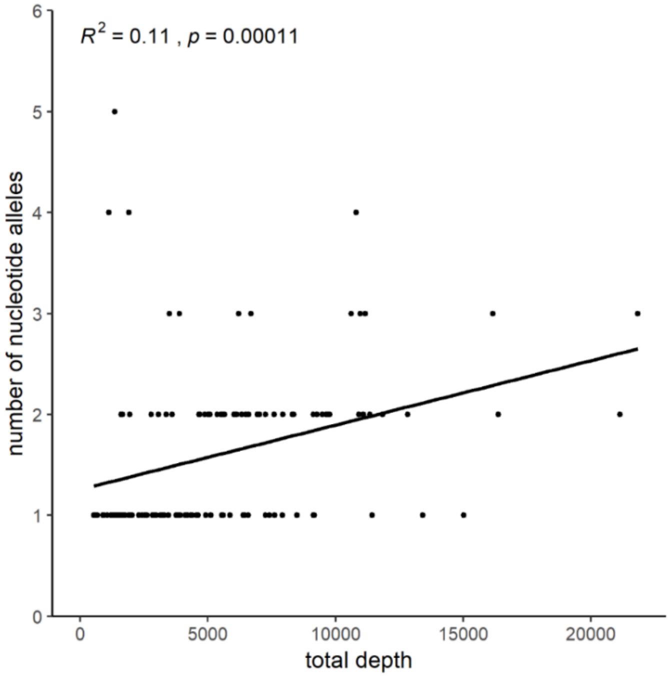

S1 Fig. Correlation between sequencing depth and the number of nucleotide alleles identified in each sample.

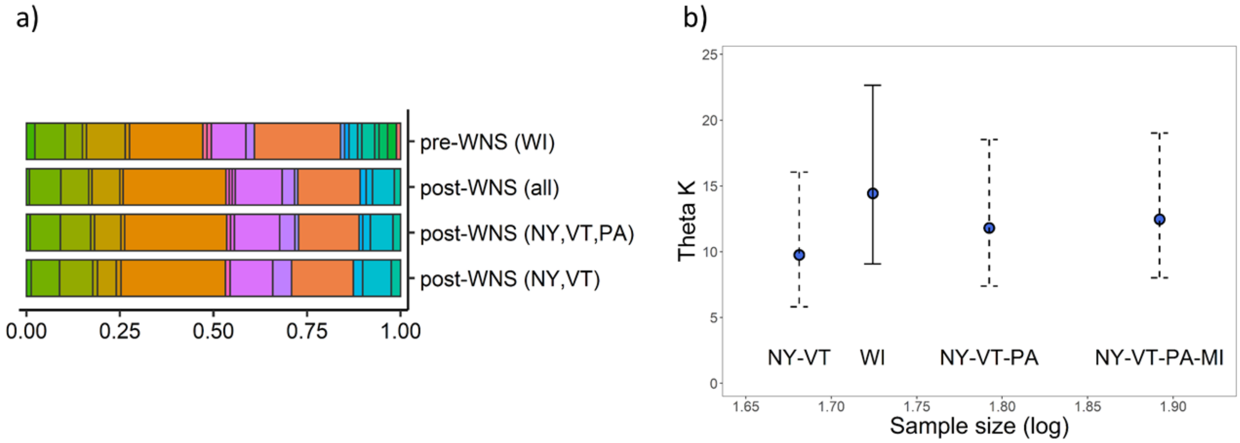

S2 Fig. Comparison of a) amino acid allele percentage and b) nucleotide allelic richness (Theta K) across populations. Error bars of Theta K show 95% confidence intervals and line types of error bars indicate WNS infection status of the corresponding sites: the pre-WNS population in solid lines and post-WNS populations in dashed lines. The pre-WNS population is composed of 3 sampling sites in WI. The post-WNS populations are composed of all sampling sites exposed to Pd (NY, VT, PA, MI), or excluding the one-year exposed MI site, or excluding both MI and PA which had a small sample size. In all groupings of populations, components of amino acid alleles were very similar and the estimation of Theta K showed no significant differences, although both data indicated relatively higher MHC diversity in the pre-WNS population.

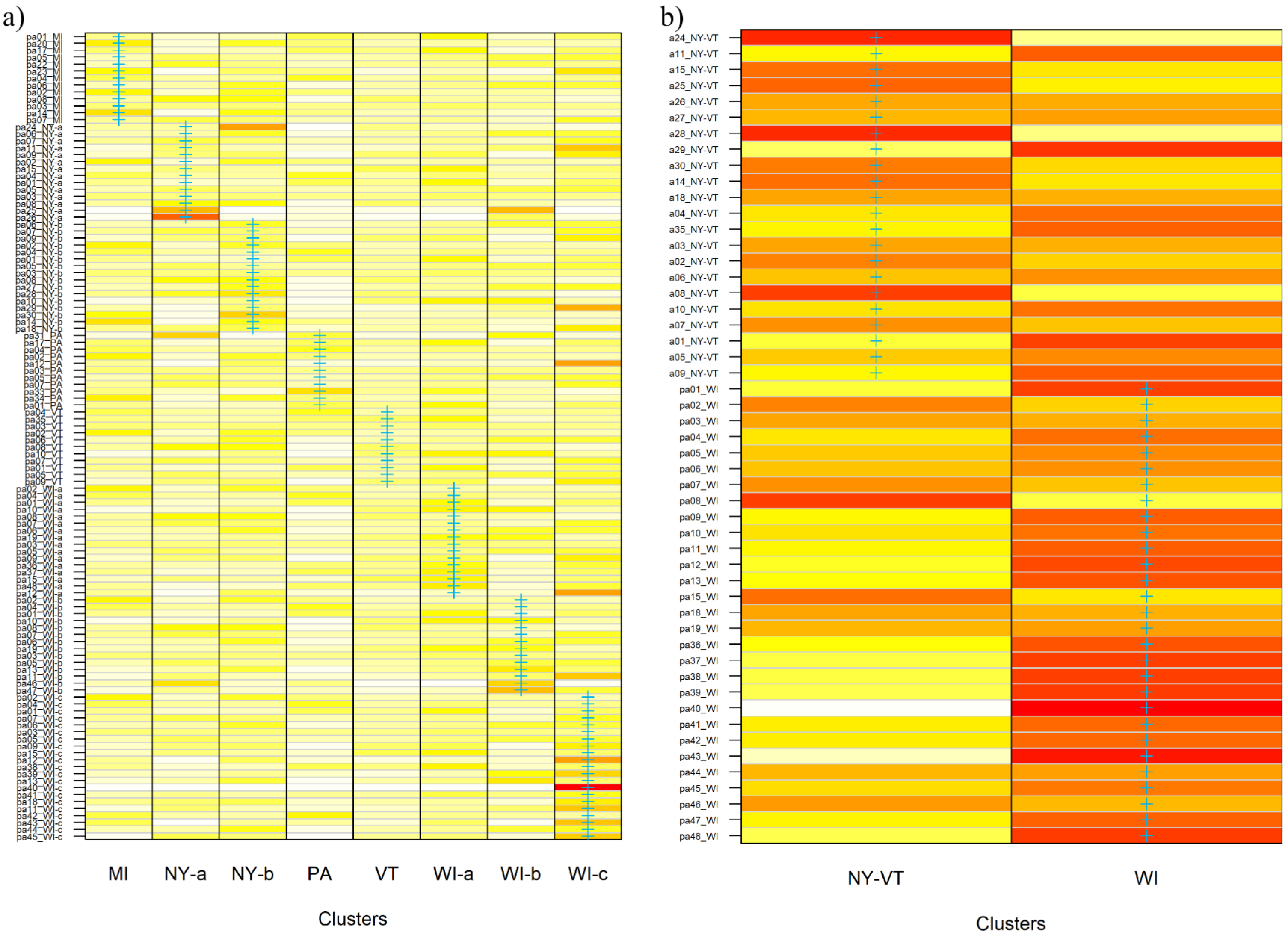

S3 Fig. DAPC heat map showing posterior probabilities of allele membership to their original clusters assigned based on a) the sampling sites or b) the pre- (WI) and post-WNS (NY, VT) populations. Cell colors reflect the probabilities (red=1, white=0) and blue crosses represent the prior clustering assignments. Reassignment to the pre- and post-WNS groups was relatively better but still had low probabilities of success (see in the main text), indicating high MHC allele admixture in the studied region.
