# Supplementary material for: Major histocompatibility complex variation is similar in little brown bats before and after white‐nose syndrome outbreak
Source: Ecol Evol. 2020 Aug 31;10(18):10031–43. doi: 10.1002/ece3.6662 (PMC7520216; doi:10.1002/ece3.6662)
Supplement: Supplementary file 2 — Tables S3‐S4 [file ECE3-10-10031-s002.pdf]

Supplementary information for Yi et al. 2020

MHC variation is similar in little brown bats before and after white-nose syndrome outbreak

The AMPtk pre-processing methods and the genotyping results.

The AMPtk pipeline for NGS data processing (https://amptk.readthedocs.io/en/latest/) was used to select reads with valid barcodes and both forward and reverse primer sequences. Primers and barcodes were trimmed in the demultiplexed reads, which were further filtered by the python script "mhc_dada2.py" (available on GitHub https://github.com/nextgenusfs/mhc_cluster). This script was specifically written to process MHC *DRB* exon 2 amplicons using the following steps: a modified DADA2 package (Callahan et al. 2016. Nature Methods https://www.nature.com/articles/nmeth.3869) to filter data based on quality, the DADA2 pipeline to denoise data and parse the output, the MHC DRB2 Hidden Markov Model (HMM) to filter reads based on translation sequences, and the final clustering (99% similarity) of reads into operational taxonomic units (OTUs, i.e. variant sequences). The OTUs were output together with a summary table of their counts in each successfully processed sample.

The AMPtk method successfully pre-processed 137 samples, and 132 of them were successfully genotyped using the CNV-DOC method (see in the main text), resulting in 50 nucleotide alleles and 37 amino acid alleles. Nucleotide alleles identified in the AMPtk and jMHC (see in the main text) pre-processed datasets were very similar in terms of allele numbers (Table S3) and DNA sequences (Table S4), suggesting robustness of our genotyping methods. Thirty-seven nucleotide sequences were identified as putative alleles in both datasets; however, 12 of the 13 nucleotide alleles that were only identified in the AMPtk pre-processed dataset had shifted reading frames (not in lengths of 283 ± 3N bp, Table S4) and thus were unlikely to be true alleles.

Sequences of the identified nucleotide alleles are available on the Dryad Digital Repository (https://doi.org/10.5061/dryad.76hdr7ssq).

Table S3. Summary and comparison of genotyping results from the AMPtk and jMHC pre-processed datasets.

	AMPtk				jMHC			
**Sampling site (WNS status)**	**n**	**I_N (R_N)**	**A_N**	**U_N**	**n**	**I_N (R_N)**	**A_N**	**U_N**
MI (1-year post)	17	1.59 (1-3)	19	4	16	1.31 (1-2)	13	3
NY-a (post)	14	1.71 (1-3)	15	1	13	1.69 (1-3)	14	3
NY-b (post)	21	1.95 (1-4)	24	4	20	1.55 (1-2)	16	4
PA (post)	14	1.71 (1-4)	11	2	14	1.50 (1-2)	11	3
VT (post)	15	2.13 (1-4)	14	1	15	1.73 (1-4)	11	1
WI-a (pre)	17	1.88 (1-4)	22	4	16	1.50 (1-3)	16	3
WI-b (pre)	15	1.33 (1-3)	15	0	15	1.27 (1-3)	14	2
WI-c (pre)	19	2.21 (1-6)	23	8	22	2.14 (1-5)	21	8
**Total**	132	1.83 (1-6)	50		131	1.61 (1-5)	45	

n: number of genotyped individuals; I_N (R_N): average (range) number of nucleotide alleles per individual; A_N: number of different nucleotide alleles from the site; U_N: number of unique nucleotide alleles from the site.

Table S4. Comparison of the identified nucleotide alleles from the AMPtk and jMHC pre-processed datasets.

	jMHC	AMPtk	length
**Identical alleles**	pa01	iSeq1	283
	pa02	iSeq3	283
	pa03	iSeq9	283
	pa04	iSeq5	283
	pa05	iSeq4	283
	pa06	iSeq7	283
	pa07	iSeq6	283
	pa08	iSeq2	283
	pa09	iSeq8	283
	pa10	iSeq12	283
	pa11	iSeq11	283
	pa12	iSeq15	283
	pa13	iSeq29	283
	pa14	iSeq30	283
	pa15	iSeq13	283
	pa17	iSeq28	283
	pa18	iSeq33	283
	pa19	iSeq16	283
	pa20	iSeq50	283
	pa22	iSeq48	283
	pa23	iSeq53	283
	pa26	iSeq45	283
	pa28	iSeq38	283
	pa29	iSeq42	283
	pa30	iSeq35	283
	pa31	iSeq41	283
	pa34	iSeq47	283
	pa35	iSeq27	283
	pa36	iSeq21	283
	pa37	iSeq55	283
	pa38	iSeq24	283
	pa39	iSeq34	283
	pa40	iSeq58	283
	pa42	iSeq54	283
	pa43	iSeq51	283
	pa45	iSeq31	283
	pa48	iSeq37	283
	**jMHC**	**AMPtk**	**length**
**Unique alleles**	pa24		283
	pa25		283
	pa27		283
	pa33		283
	pa41		283
	pa44		283
	pa46		283
	pa47		283
		iSeq167	295
		iSeq10	291
		iSeq17	291
		iSeq49	291
		iSeq18	290
		iSeq26	290
		iSeq62	290
		iSeq84	290
		iSeq93	290
		iSeq109	290
		iSeq117	290
		iSeq245	290
		iSeq19	284
